# Surgical site infections following emergency abdominal surgery: a prospective cohort study

**DOI:** 10.4314/ahs.v25i1.4

**Published:** 2025-03

**Authors:** Mert Adali

**Affiliations:** Bursa Yuksek İhtisas Training and Research Hospital of Health Sciences University, General Surgery Clinic, Bursa, Turkey

**Keywords:** Abdominal surgery, ASEPSIS, Emergency surgery, Surgical site infection

## Abstract

**Background:**

Surgical site infection (SSI) is of major concern in surgical patients due to increased morbidity and mortality, prolonged hospital stay, increased need for intensive care, increased costs and hospital readmissions. The main objective of this study was to define the incidence and risk factors associated with SSI in patients undergoing emergency abdominal surgery.

**Methods:**

This is a prospective cohort study. Patients over the age of 18 who underwent emergency abdominal surgery were included in this study. [ma1]Patients under 18 years of age, who underwent elective surgery and who underwent procedures outside the abdomen were excluded from the study. Wound assessments were performed according to ASEPSIS score.

**Results:**

A total of 130 patients were included in the study. The incidence of SSI was found to be 10.0%. The prevalence of SSI was 27.5% (8/29) in patients operated for intestinal obstruction and 4.9% (5/101) in other patients (p=0.001). [ma2]Predictors[ma3] of SSI were open surgical approach, contaminated/dirty wound group, intestinal obstruction, colorectal surgery, increasing incision length[ma4] and age.

**Conclusion:**

This study identified several independent risk factors for SSI after emergency abdominal surgery that need to be addressed. [ma5]

## Introduction

SSI is defined as infections observed at the surgical site that may develop within 30 or 90 days after surgery (the day of surgery is considered the first day)^1^. SSI is of great importance for surgical patients because of increased morbidity and mortality, prolonged hospital stay, increased need for intensive care, increased costs and hospital readmissions^2^. The development of SSI is multifactorial and may be associated with risk factors such as patient age, comorbidities, smoking status, obesity, malnutrition, immunosuppression, malignancy and wound contamination class^3^. It is one of the most common healthcare-associated infections, occurring following 1-3% of all surgical procedures^4^. SSI rates are much higher in abdominal surgery than in other types of surgery and have been found to be 15-25% depending on the level of contamination in various prospective studies^4^-^6^. Emergency surgery is a risk factor for SSI because many strong risk factors such as contaminated and dirty wounds, prolonged operative time, patient comorbidities and high American Society of Anaesthesiologists (ASA) score are often present in this type of surgery^2^. There[ma6] are various scoring systems to objectively assess SSI. The ASEPSIS score is an objective wound scoring system based on specific clinical findings that provides a quantitative analysis to determine the severity of SSI^7^. With the increasing incidence of SSIs and associated morbidity, various studies have investigated ways to improve the management of patients during the recovery period to prevent SSIs^8^,^9^. The main objective of this study was to define the incidence and risk factors associated with SSI in patients undergoing emergency abdominal surgery.

## Methods

### Study desing and setting

The study was designed as a prospective cohort study. A total of 130 patients who underwent emergency abdominal surgery between January 2023 and June 2023 at the General Surgery Clinic of Health Sciences University Bursa High Speciality Training and Research Hospital were included in the study. Prior to the start of the study, approval was obtained from the Clinical Research Ethics Committee of the hospital with decision number 2011-KAEK-25 2022/10-04 dated 05.10.2022.

### Data collection procedure

Patients over 18 years of age who underwent emergency abdominal surgery for acute abdomen were included in the study. Patients were excluded from the study if they were under 18 years of age, underwent elective surgery or underwent surgery other than abdominal surgery. The decision to operate was made by the physician on duty that day and the patient was operated by the same physician. Clinical procedures for SSI prophylaxis were: administration of prophylactic antibiotics before incision (cefazolin 1-2 g IV, cefazolin 1-2 g IV + metronidazole 500 mg IV or ciprofloxacin 400 mg IV), skin antisepsis with 10% povidone iodine. Patients were identified on the first postoperative day and a patient follow-up form was created for inclusion in the study. All patients enrolled in the study were informed about all study procedures and the processing of personal data. Informed consent was then obtained and signed. In the case of normal healing, patients were informed of wound complications after discharge. Patients were followed prospectively for 30 days. If wound complications occurred during hospitalisation or after discharge, the wound was assessed in detail. If bacterial infection was suspected, a wound swab was taken for culture. All possible adjuvant procedures (e.g. surgical drainage, negative pressure wound therapy) were decided according to the opinion of the attending physician. During clinical follow-up, demographics, comorbidities, medical history, surgical site characteristics, incision type and length, drain use, body mass index (BMI), hospital stay, and ASEPSIS score were calculated and recorded. All patient data were recorded on the patient follow-up form. ASEPSIS scores were calculated daily for all patients. Surgical wounds were evaluated by calculating the modified ASEPSIS score, which was extended to 30 days post-operatively. The[ma7] ASEPSIS score is calculated by examining and scoring the incision in the first 7 days after surgery. The modified ASEPSIS score is calculated by examining the incision status in the first 30 days after surgery.

The highest score calculated during the hospitalisation period was recorded as the patient's ASEPSIS score. The score was calculated according to the proportion of the wound affected by serous exudate, erythema, purulent exudate and deep tissue separation.[ma8] Additional points were awarded for antibiotic treatment, abscess drainage under local anaesthesia, wound debridement under general anaesthesia, bacterial isolation and hospitalisation for 14 days [7]. An ASEPSIS score of 20 and above was considered a SSI. Patients were divided into two groups as SSI (+) and SSI (-). The evaluated parameters were compared between the two groups.

### Data management and analysis[-ma9]

IBM SPSS Statistics for Windows, version 21.0 (IBM Corp. Armonk, NY: USA. Released 2012) was used for statistical analyses. Descriptive statistics were expressed as mean ± standard deviation (minimum - maximum), median and range and/or interquartile range (IQR) for numerical variables, while categorical variables were expressed as number of cases and (%). The Kolmogorov-Smirnov test was used to test the normality of the data distribution. Levene's test was used to determine whether the assumption of homogeneity of variances was met. The Mann-Whitney U test was used to assess the significance of the difference between groups for continuous numerical variables for which the statistical assumptions of parametric testing were not met. Spearman correlation analysis was used to assess relationships between non-parametrically distributed data. Chi-square and Fisher's exact test were used to analyse the relationship between categorical variables[ma10]. p<0.05 was considered statistically significant. Results were presented with 95% confidence interval.

## Results

### Demographic and clinical characteristics[ma11]

The study included 130 patients who underwent emergency abdominal surgery in our clinic. The gender distribution of the patients was 84 (64.6%) males and 46 (35.4%) females. The median age of the patients was 42 years. When the surgical wounds were categorised, clean-contaminated wounds were most commonly observed in 77 (59.2%) patients, followed by contaminated wounds in 26 (20%) patients. The most common type of incision was the full midline incision (46.9%). Intra-abdominal drain was used in 71.5% of the patients. There were a total of 14 (10.8) patients with a history of treatment for any system malignancy[ma12][ma13]. SSI was observed in 13 (10%) patients and incisional[ma14][ma15] negative pressure wound therapy was applied in 11 (8.5%) patients. Intestinal obstruction was observed in 29 (22.3%)patients. A[ma16][ma17] total of 18 (13.8%) gastrointestinal (GI) perforations were observed, including 13 upper GI perforations and 5 colon perforations (Table 1).

**Table 1:** Distribution of clinical and demographic characteristics

Age (year), median IQR (25-75)		42 (30-62)
**Gender (%)**	Male	84 (64.6[ma18])
	Female	46 (35.4)
**Comorbidity (%)**	HT	23 (17.7)
	DM	6 (4.6)
	CHD	11 (8.5)
	COPD	6 (4.6)
	Other	29 (22.3)
**Malignancy (%)**	Yes	14 (10.8)
	No	116 (89.2)
**Active smoking[ma19]** **(%)**	Yes	52 (40)
	No	78 (60)
**Wound Class (%)**	Clean	16 (12.3)
	Clean - contaminated	77 (59.2)
	Contaminated	26 (20)
	Dirty	11 (8.5)
**Operative procedure (%)**	Open	86 (66.2)
	Laparoscopic	44 (33.8)
**Incision (%)**	Lower midline	3 (2.3)
	Upper midline	5 (3.8)
	Full midline	61 (46.9)
	Subcostal	1 (0.8)
	Mc Burney	15 (11.5)
	Paramedian	1 (0.8)
	Laparoscopic	44 (33.8)
**Subcutaneous drain (%)**	Yes	7 (5.4)
	No	123 (94.6)
**Intra-abdominal drain (%)**	Yes	93 (71.5)
	No	37 (28.5)
**Intestinal Obstruction (%)**	Yes	29 (22.3)
	No	101 (77.7)
**GI perforation (%)**	Yes	18 (13.8)
	No	112 (86.2)
**NPWT (%)**	Yes	11 (8.5)
	No	119 (91.5)
**SSI (%)**	Yes	13 (10)
	No	117 (90)
**Incision length (cm), median IQR (25-75)**		12,5 (4-20)
**Length of hospital stay (days), median IQR (25-75)**		5 (2-7)
**BMI (Kg/m2), median IQR (25-75)**		25 (22-27)

HT: Hypertansion, DM: Diabetes mellitus, CHD: Chronic heart disease, COPD: hronic Obstructive pulmonary disease, GI: Gastrointestinal, NPWT: Negative pressure wound therapy, SSI: Surgical site infection, BMI: Body mass index

**Figure 1:**
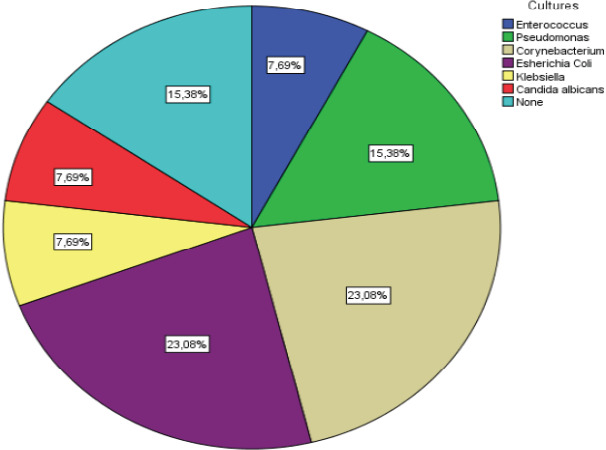
Culture results of 13 patients with SSI_[ma25]_

When patients were grouped according to the occurrence of SSI[ma21] and gender, active smoking[ma22], use of intra-abdominal drain and BMI parameters were compared between groups, no statistically significant relationship was found (p>0.05). The[ma23] parameters found to be statistically significant in terms of SSI occurrence were age, incision length, history of malignancy, operative procedure, wound class, intestinal obstruction, colorectal surgery and length of hospital stay (p<0.05). Comparisons between the groups were summarised in Table 2. Spearman correlation analysis performed to determine if there was a correlation between ASEPSIS scores and age, BMI, incision length and length of hospital stay revealed a significant positive correlation between ASEPSIS score and age, incision length and length of hospital stay [(p<0.001, r=0.392), (p<0.001, r=0.414), (p<0.001, r=0.566)] (Table 3). Microbiological culture results of 13 patients with SSI were analysed. The most common pathogens were Escherichia coli and Corynebacterium in 3 patients. These were followed by Pseudomonas which was detected in 2 patients. No growth was detected in the wound cultures of 2 patients with SSI (Figure 1)

**Table 2:** Comparison of variables according to the incidence of surgical site infection

		SSI (+) (n=13)	SSI (-) (n=117)	P value
**Age (year), median IQR (25-75)**		60 (51-73)	39 (28-61)	***.001^c^***
**Gender (%)**	Male	11 (84.6)	73 (62.4)	.136^a^
	Female	2 (15.4)	44 (37.6)	
**Comorbidity (%)**	HT	2 (15.4)	21 (17.9)	.587^a^
	DM	1 (7.7)	5 (4.3)	.476^a^
	CAD	2 (15.4)	9 (7.7)	.302^a^
	COPD	1 (7.7)	5 (4.3)	.476^a^
	Other	4 (30.8)	25 (21.4)	.485^a^
**BMI (Kg/m2), median IQR (25-75)** **Malignancy (%)**		25 (23-26)	25 (22-27)	.828 ^c^
	Yes	5 (38.5)	9 (7.7)	***.005^a^***
	No	8 (61.5)	108 (92.3)	
**Active smoking[ma24] (%)**	Yes	4 (30.8)	48 (41)	.474^b^
	No	9 (69.2)	69 (59)	
**Operative procedure (%)**	Open	13 (15.1)	73 (62.4)	***.004^a^***
	Laparoscopic	0 (0)	44 (37.6)	
**Wound Class (%)**	Clean/Clean-contaminated	6 (46.2)	87 (74.4)	***.049^a^***
	Contaminated/Dirty	7 (53.8)	30 (25.6)	
**Intra-abdominal drain (%)**	Yes	12 (92.3)	81 (69.2)	.108^a^
	No	1 (7.7)	36 (30.8)	
**Intestinal Obstruction (%)**	Yes	8 (61.5)	21 (17.9)	***.001^a^***
	No	5 (38.5)	96 (82.1)	
**GI perforation (%)**	Yes	3 (23.1)	15 (12.8)	.389^a^
	No	10 (76.9)	102 (87.2)	
**Colorectal surgery (%)**	Yes	6 (46.2)	16 (13.7)	***.009^a^***
	No	7 (53.8)	101 (86.3)	
**Incision length (cm), median IQR (25-75)**		20 (20-25)	10 (4-20)	***.000 ^c^***
**Length of hospital stay (days), median IQR (25-75)**		24 (14-37)	3 (2-7)	***.000 ^c^***

a: Fisher's Exact Test

b: Pearson Chi-Square

c: Mann-Whitney U

**Table 3:** Spearman Correlation Analysis Table of Variables

		ASEPSİS score	Age	BMI	Incision length	Length of hospital stay
**ASEPSİS**	r	1	.392	-.035	.414	.566
**score**	p		***.000***	.690	***.000***	***.000***
**Age**	r	.392	1	.249	.550	.560
	p	***.000***		***.004***	***.000***	***.000***
**BMI**	r	-.035	.249	1	.113	.104
	p	.690	***.004***		.199	.240
**Incision length**	r	.414	.550	.113	1	.736
	p	***.000***	***.000***	.199		***.000***
**Length of hospital stay**	r	.566	.560	.104	.736	1
	p	***.000***	***.000***	.240	***.000***	

r: Correlation coefficient, BMI: Body mass index

## Discussion

The study showed that the incidence of SSI was 10.0% in patients who underwent emergency abdominal surgery in a single unit during the period evaluated. When analysing similar studies, Li et al^10^ found this rate to be 7.5% and Papadopulos et al^11^ found it to be 18.7%. The incidence of postoperative SSI is generally higher in patients undergoing colorectal surgery compared to other abdominal surgery^12^. According to the results of this study, the incidence of SSI was higher in the emergency colorectal surgery group compared to other emergency abdominal surgery procedures (p=0.009). It is difficult to prepare the bowel for emergency surgery, and the bowel contents spill out and contaminate the surgical field. This explains the high risk of SSI in emergency colorectal surgery patients. In the study, the risk of SSI was found to be higher in the patient group with intestinal obstruction (p=0.001). Dilatation of the intestinal lumen compromises the intestinal barrier function and bacteria are easily displaced outside the intestinal lumen, increasing the risk of SSI. This study found no significant difference in the incidence of SSI[ma26] in the group of patients with gastrointestinal perforation compared with the group without perforation. Li et al.^10^ also found in their study that gastrointestinal perforation was not a risk factor for SSI. Gastrointestinal perforation usually occurs in the upper gastrointestinal tract, which has a relatively low bacterial load. The risks of SSI are reduced by adequate irrigation during surgery and appropriate antibiotic therapy after surgery. [ma27] Previous studies have shown that diabetes and preoperative hyperglycaemia are risk factors for SSI^13^. In this study, all the comorbidities we examined, including DM and some other known risk factors for SSI such as BMI, DM and smoking, were not statistically significant. This may be due to the sample size and diversity of cases in our study. In[ma28][ma29] our study, a significant correlation was found between the length of hospital stay and SSI. The median length of hospital stay was 24 (14-37) days in the group with SSI and 3 (2-7) days in the group without SSI. Many previous studies have reported similar findings regarding prolonged hospital stay in patients with SSI^14^,^15^.

Prolonged hospital stay is a condition that increases the risk of SSI. In addition, the development of SSI is a reason for prolonging the hospital stay. When we analysed the data from our study, we found a positive correlation between the ASEPSIS score and the length of hospital stay. It was not possible to establish a cause and effect relationship in accordance with the data obtained. In this study, age, history of malignancy, open surgery, contaminated/dirty wound group were found to be factors increasing the risk for SSI. Several studies have shown that open surgery is a risk factor for SSI compared to laparoscopic surgery^12^,^16^,^17^. Laparoscopic surgery uses a small incision and provides a wide field of view with less damage to surrounding tissue, reducing the risk of SSI. Several studies have shown that the contaminated/dirty wound group is a risk factor for SSI compared with the clean/contaminated group^11^,^18^. Unlike similar studies, this study analysed the relationship between incision length[-ma30] and SSI and found a statistically significant relationship (p<0.001). Spearman correlation analysis also showed a positive correlation between incision length and ASEPSIS score. A multicentre study showed that increasing age up to 65 years was associated with an increased risk of SSI^19^. Another study found that increasing age increased the risk of SSI^11^. In this study, a significant positive correlation was found between age and ASEPSIS score[ma31]. The results of the study suggest that increasing age increases the risk of SSI and are consistent with the literature.

This[ma32] is a prospective study in which all data were collected regularly and systematically. The ASEPSIS score was used to assess SSI. As a reliable and standardized tool for measuring the severity of infections, the ASEPSIS score enhances the validity and reliability of the study results. This study has several limitations. The duration of the study was short and included only patients who underwent emergency abdominal surgery between January - 2023 and June - 2023. The study included different types of patients and different methods of surgery. More reliable results may be obtained with longer follow-up and a larger number of patients. The sample size was small and multivariate analysis was not possible. There is a need for larger and multi-centre trials on this topic.
